# Factors influencing U.S. women’s interest and preferences for breast cancer risk communication: a cross-sectional study from a large tertiary care breast imaging center

**DOI:** 10.1186/s12905-024-03197-7

**Published:** 2024-06-21

**Authors:** Jessica D. Austin, Emily James, Rachel L Perez, Gina L. Mazza, Juliana M. Kling, Jessica Fraker, Lida Mina, Imon Banerjee, Richard Sharpe, Bhavika K. Patel

**Affiliations:** 1 Department of Quantitative Health Sciences, Division of Epidemiology, Mayo Clinic, 13400 E. Shea Blvd, Scottsdale, AZ 85259 USA; 2 Mayo Clinic College of Medicine of Medicine and Science, Mayo Clinic, 5777 E Mayo Blvd, Phoenix, AZ 85054 USA; 3 Department of Quantitative Health Sciences, Division of Clinical Trials and Biostatistics, Mayo Clinic, 13400 E. Shea Blvd, Scottsdale, AZ 85259 USA; 4 Women’s Health Internal Medicine, Department of Internal Medicine, Mayo Clinic, 13400 E. Shea Blvd, Scottsdale, AZ 85259 USA; 5 Department of Internal Medicine, Division of Medical Oncology, Mayo Clinic, 5777 E Mayo Blvd, Phoenix, AZ 85054 USA; 6 Department of Diagnostic Radiology, Mayo Clinic, 5777 E Mayo Blvd, Phoenix, AZ 85054 USA

**Keywords:** Breast cancer risk assessment, Women’s preferences, Health communication, Mammography

## Abstract

**Background:**

Breast imaging clinics in the United States (U.S.) are increasingly implementing breast cancer risk assessment (BCRA) to align with evolving guideline recommendations but with limited uptake of risk-reduction care. Effectively communicating risk information to women is central to implementation efforts, but remains understudied in the U.S. This study aims to characterize, and identify factors associated with women’s interest in and preferences for breast cancer risk communication.

**Methods:**

This is a cross-sectional survey study of U.S. women presenting for a mammogram between January and March of 2021 at a large, tertiary breast imaging clinic. Survey items assessed women’s interest in knowing their risk and preferences for risk communication if considered to be at high risk in hypothetical situations. Multivariable logistic regression modeling assessed factors associated with women’s interest in knowing their personal risk and preferences for details around exact risk estimates.

**Results:**

Among 1119 women, 72.7% were interested in knowing their breast cancer risk. If at high risk, 77% preferred to receive their exact risk estimate and preferred verbal (52.9% phone/47% in-person) vs. written (26.5% online/19.5% letter) communications. Adjusted regression analyses found that those with a primary family history of breast cancer were significantly more interested in knowing their risk (OR 1.5, 95% CI 1.0, 2.1, *p* = 0.04), while those categorized as “more than one race or other” were significantly less interested in knowing their risk (OR 0.4, 95% CI 0.2, 0.9, *p* = 0.02). Women 60 + years of age were significantly less likely to prefer exact estimates of their risk (OR 0.6, 95% CI 0.5, 0.98, *p* < 0.01), while women with greater than a high school education were significantly more likely to prefer exact risk estimates (OR 2.5, 95% CI 1.5, 4.2, *p* < 0.001).

**Conclusion:**

U.S. women in this study expressed strong interest in knowing their risk and preferred to receive exact risk estimates verbally if found to be at high risk. Sociodemographic and family history influenced women’s interest and preferences for risk communication. Breast imaging centers implementing risk assessment should consider strategies tailored to women’s preferences to increase interest in risk estimates and improve risk communication.

**Supplementary Information:**

The online version contains supplementary material available at 10.1186/s12905-024-03197-7.

## Background

Breast cancer remains the most common malignancy in the United States (U.S.) with wide variation in incidence and mortality. This variation is attributable to a myriad of factors such as biological sex, age, reproductive history, hormone use, family history, genetic mutations, breast density, obesity, and alcohol intake, each alone explaining a modest proportion in variation in risk [1–4]. Evolving guideline organizations [5–7], including the National Comprehensive Cancer Network, American College of Radiology, and American Cancer Society, recommend formal breast cancer risk assessment (BCRA) starting between 25 and 30 years of age to guide those at increased risk to appropriate risk-reduction care. Risk-based approaches to breast cancer screening have the potential to decrease harms (i.e., false-positive, overdiagnosis) associated with current age-based approaches and may improve early detection in high-risk women leading to improvements in survival rates and quality of life [8–10].

Several validated models exist that utilize a combination of women’s patient-reported information and medical records data to accurately quantify a woman’s lifetime, 10-year or 5-year risk of developing breast cancer [11], and are increasingly used at mammography screening facilities to facilitate guideline-recommended risk-reduction care. Implementation of these models in clinical settings has shown to significantly improve identification of individuals at high-risk for breast cancer [12–15]; but have not led to the uptake guideline recommended preventive care including supplemental screening, genetic testing/counseling, or risk reducing medications for those at increased risk [13, 15–20] While multifaceted, these findings may be partially explained by ineffective risk communication. Integration of BCRAs into electronic health records lend itself to delivering written communication to clinicians and women via clinical reports or patient portals. Yet, these communications of breast cancer risk results have not led to changes to women’s risk perceptions or uptake of recommended care [21, 22] Moreover, implementation of risk-based approaches should be accepted and supported by women [23]. Limited prior studies suggest that women welcome risk-based screening [24, 25], but few include perspectives of women in the U.S.

As efforts to implement BCRAs increase, several questions remain regarding women’s interest in knowing their risk and how best to communicate breast cancer risk estimates in a manner that aligns with their preferences. Effective risk communication is essential for helping women understand their vulnerability to a disease [26, 27]. Yet, most women are unaware of or misperceive their breast cancer risk [28, 29]. These misperceptions can have harmful consequences, including emotional distress and missed opportunities to utilize guideline recommended preventive care for those at increased risk [30, 31] Prior efforts to improve risk communication have largely been tasked to clinicians to address gaps in clinician and organizational barriers to implementation [32–34]. While important, successful implementation of BCRA requires an understanding of women’s preferences for communication to optimize transfer of risk knowledge and recommendations. This study aims to characterize and identify factors associated interest in and preferences for breast cancer risk communication among a large cohort of U.S. women undergoing mammography screening at a large breast imaging clinic.

## Methods

This is a cross-sectional, quality improvement, survey study on a convenient sample of 1221 women presenting for screening mammography at the Mayo Clinic in Arizona (MCA) Breast Imaging Clinic between January 2021 and March 2021. The Breast Imaging Clinic provides approximately 14,000 screening mammograms annually, including no-cost screening to underserved populations through community-based partnerships. Informed consent was not required for this study as it was deemed exempt from ethics approval by the Mayo Clinic Institutional Review Board.

### Data collection

A paper survey was administered at the time of women’s mammography screening appointment. Adapted from prior assessments [35, 36], the 18-item survey (see Appendix) assessed sociodemographic characteristics, known breast cancer risk factors, and included items assessing if the women were ever provided a personal risk estimate, interest in knowing personal risk, and preferences for receiving and communicating risk information.

#### Interest in knowing breast Cancer risk

Interest in knowing one’s personal risk for breast cancer is the primary outcome of this analysis. Women indicated their level of agreement on a 5-point scale (‘Strongly agree’ to ‘Strongly disagree’) to the following item: *“I am interested in knowing my risk for breast cancer”.* For this analysis, responses were dichotomized as ‘interested’ (‘strongly agree’/’agree’) and ‘neutral’/’uninterested’ (‘neither agree nor disagree’/’disagree’/ ‘strongly disagree’). Those who did not respond to this item were excluded from the analysis (*n* = 10).

#### Preferred mode of risk communicatio

Women were presented with a hypothetical scenario in which they were at high risk for breast cancer and asked how they prefer to receive this information and in how much detail [35, 36]. Women’s preferences for receiving information about their breast cancer risk was assessed using a single item: *“If you are found to be at high risk of breast cancer, how would you prefer to receive the result of your estimated breast cancer risk?”.* Women had the ability to select multiple options including ‘Face-to-face meeting with the health professional who ordered the mammogram’, ‘Telephone call from the health professional who ordered the mammogram’, ‘Face-to-face meeting with the radiologist who interpreted your mammogram’, ‘Telephone call from the radiologist who interpreted your mammogram’, ‘Face-to-face meeting with a breast risk practitioner’, ‘Telephone call from a breast risk practitioner’, ‘Mailed letter accompanying your annual mammogram result’, ‘Mailed letter separate from your mammogram result’, ‘E-mailed copy separate from your mammogram result’, ‘View the result through Patient Online Services (MyChart)’, and ‘Referral to a high-risk breast center’. For this analysis, responses were categorized as face-to-face with a health care professional, telephone call from a healthcare professional, mailed letter, electronic communication, or referral.

#### Preferred level of detail for risk communication

Women were also asked how much detail they prefer to receive if they were considered high risk for breast cancer. Response options were ‘less detailed (for example, “your calculated breast cancer risk was high and you may need further testing”)’, ‘moderate detail (for example, “your calculated breast cancer risk was greater than 20% and you may need further testing”)’, ‘very detailed (for example, “your calculated breast cancer risk was 26%, which is considered high risk, and you may need further testing”)’, and ‘I would not like my risk to appear in my radiology report’. Responses were dichotomized as ‘yes’ (‘Very detailed’) or ‘no’ (‘Moderate detail’, ‘Less detail’, ‘No detail’) to wanting their exact risk estimate.

### Analysis

To align with guideline recommendations for initiating breast cancer screening for women at average risk, we excluded women under the age of 40 (*n* = 34). We also excluded women with a history or unknown history of breast cancer (*n* = 53) or a known genetic mutation for breast cancer (*n* = 5) since their interest and preferences for risk communication likely differ from those without a cancer diagnosis. The final sample size for this analysis was 1119 women. Summary statistics were calculated to describe the distribution of key variables. We examined differences in interest in knowing one’s breast cancer risk estimate (interested vs. neutral/not interested) by sociodemographic, breast cancer risk factors, and mammography screening history using Fisher’s exact test. We estimated two multivariable logistic regression models to identify sociodemographic and breast cancer risk factors predictive of one’s interest in (1) knowing their breast cancer risk and (2) knowing their exact breast cancer risk estimate. To provide supplemental information, these multivariable logistic regression models were also estimated while excluding women who reported ever having received an estimate of their breast cancer risk. Results from the multivariable logistic regression models are reported using adjusted odds ratios (OR) and 95% Wald confidence intervals (CI). All analyses were performed using SAS 9.4 with p-values < 0.05 considered statistically significant.

## Results

A summary of patient characteristics is provided in Table 1. Most women were 60 years of age or older (51.2%), self-identified as white (86.8%) and non-Hispanic (92.7%), with greater than a high school education (93.1%). In addition, 81.1% reported no primary family history of breast cancer, 77.2% reported no prior breast biopsy, and 83.5% report receiving a mammogram annually.

**Table 1:**
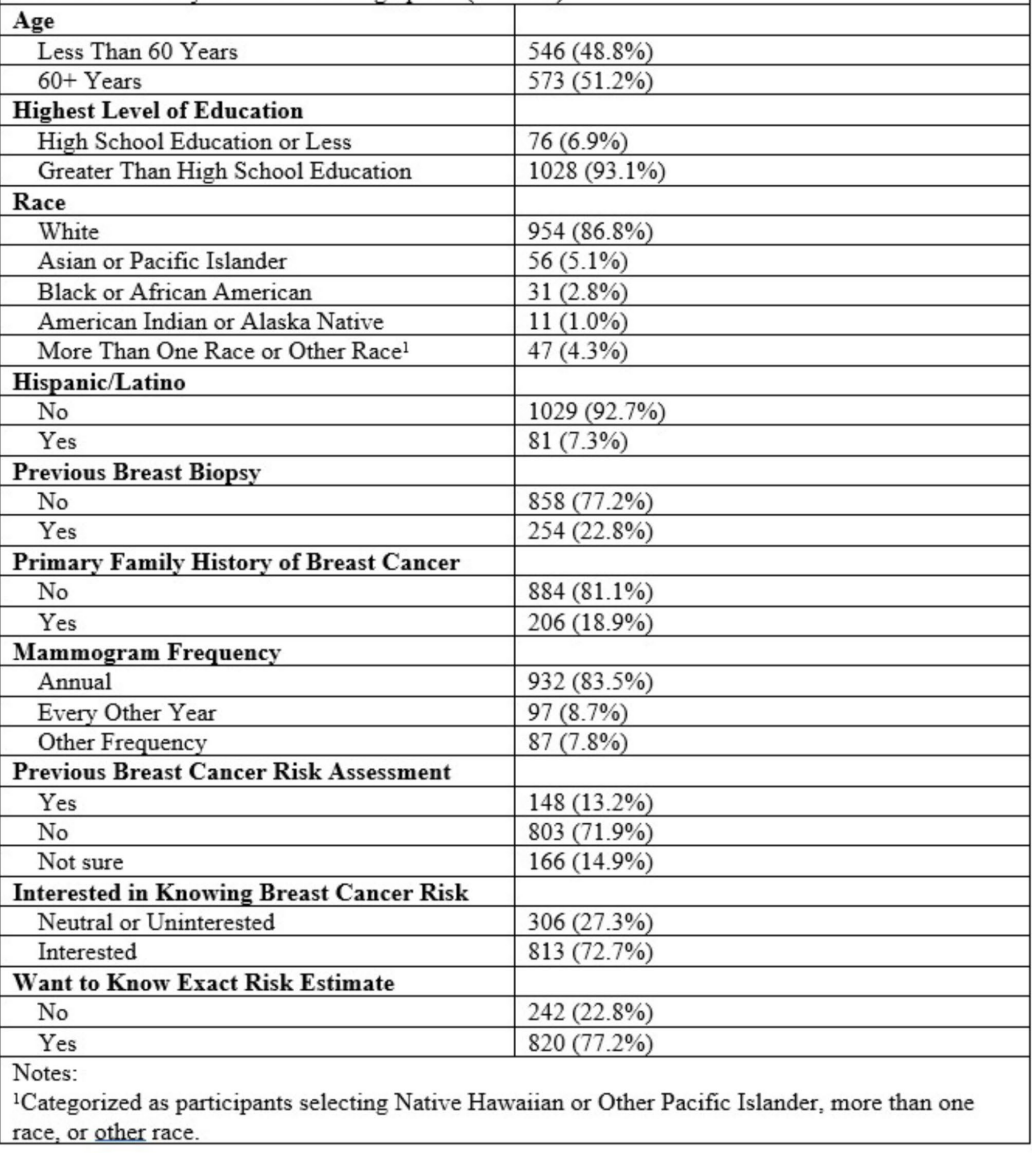
Summary of participant characteristics (*N*=1119)

### Interest in knowing breast cancer risk

**Fig. 1:**
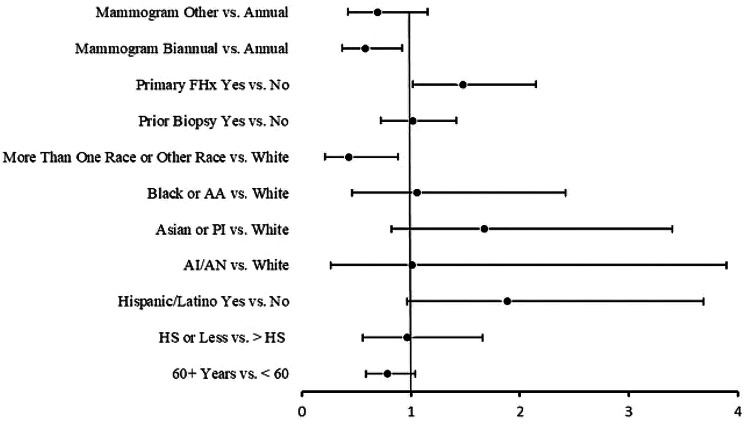
Forest plot of the odds ratio and 95% confidence intervals of factors predicting interest in knowing one’s personal risk for breast cancer for the entire sample (*N*=1058). Abbreviations: FHx, Family History; AA, African American; PI, Pacific Islander; AI/AN, American Indian or Alaskan Native; HS, High School

Overall, 72.7% of women were interested in knowing their risk for breast cancer though only 13.2% reported ever being provided their personal breast cancer risk. Interest in knowing one’s personal risk differed by mammography frequency, with women receiving mammograms annually reporting higher interest in knowing their risk (Fisher’s exact *p* = 0.01; Table 2). Results from the multivariable logistic regression analysis for our entire sample (see Fig. 1) show that women with a primary family history of breast cancer were significantly more interested in knowing their risk compared to women without a primary family history (OR 1.5; 95% CI 1.0, 2.1; *p* = 0.04), while women categorized as “more than one race or other” were significantly less interested in knowing their risk compared to women identifying as White (OR 0.4; 95% CI 0.2, 0.9; *p* = 0.02). Mammography frequency was not significantly associated with interest in knowing one’s breast cancer risk when controlling for all other variables in the model. These findings remained in supplementary analyses excluding women who were ever provided their risk estimates. Additionally, women 60 years of age and older were significantly less interested in knowing their breast cancer risk compared to women under the age of 60 (OR 0.7; 95% CI 0.5, 0.9; *p* = 0.02) after excluding women ever provided their risk estimate.

**Table 2:**
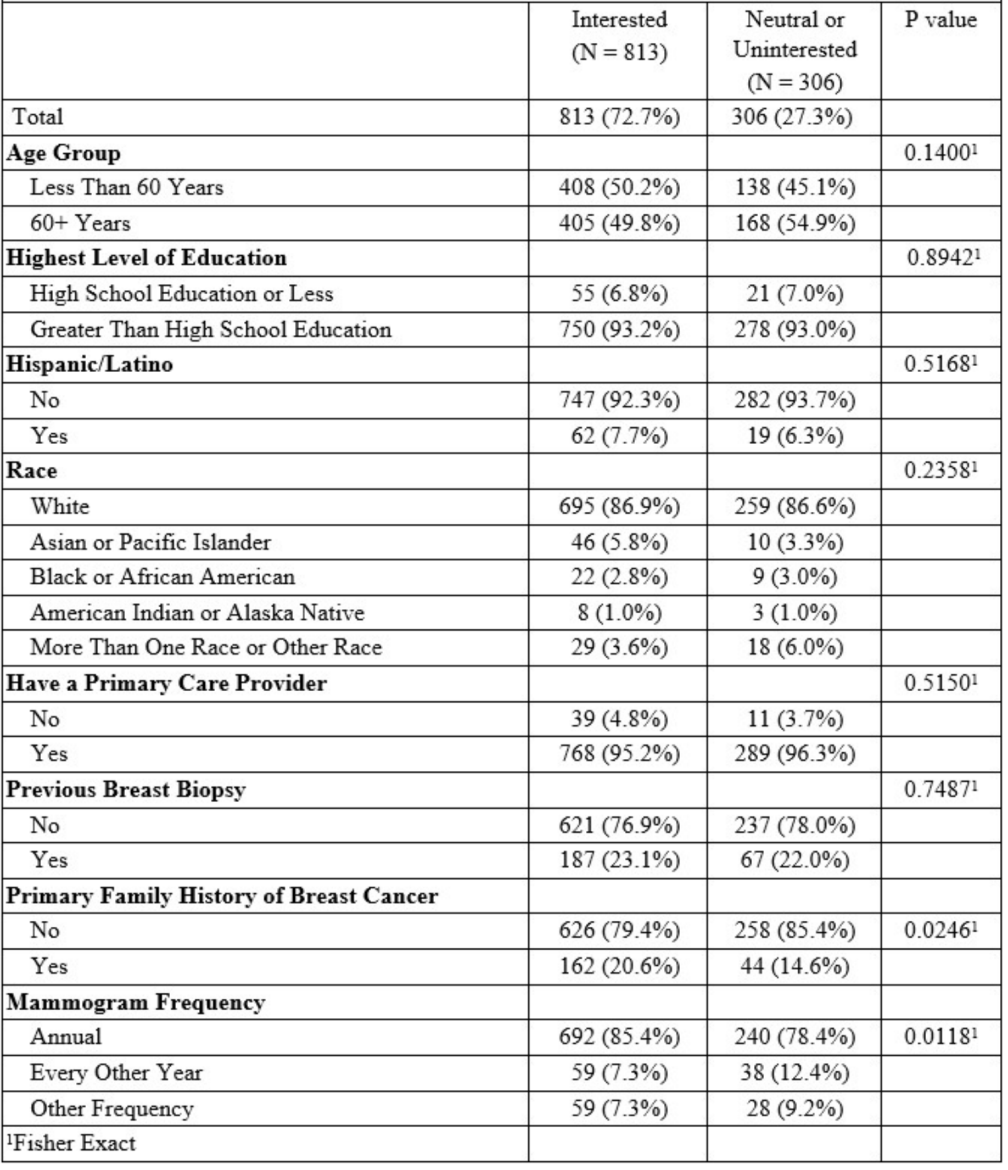
Differences in interest in knowing breast cancer risk by participant characteristics.

### Preferred mechanism for risk communication

**Fig. 2:**
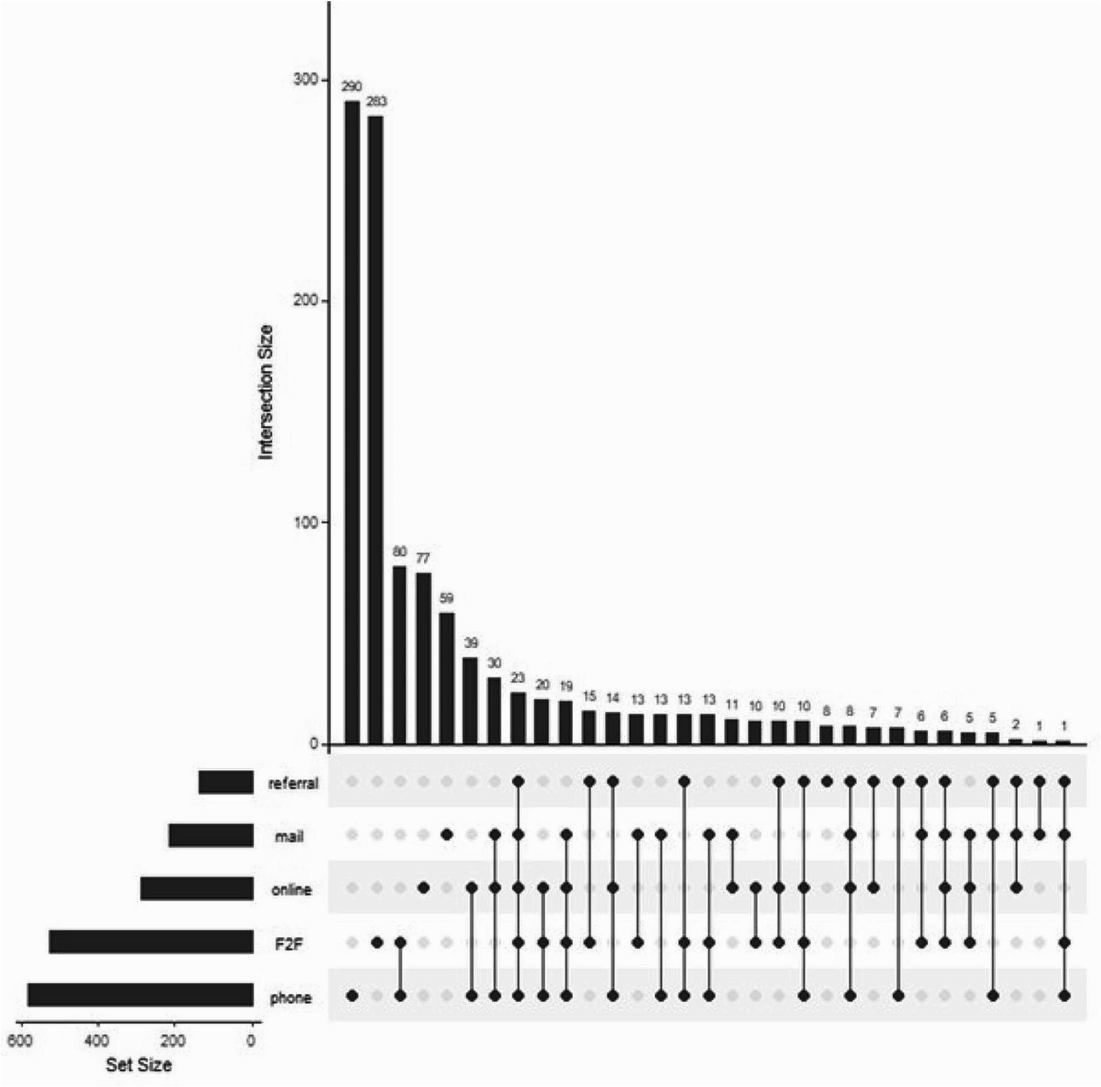
UpSet plot showing the number of women endorsing each combination of preferences for receivingbreast cancer risk estimates. Notes: 21 patients made no selection regarding their preferences. F2F=face-to-face.

Figure 2 describes preferences for risk communication for our entire sample. If considered to be at high risk for breast cancer, 52.9% would prefer to receive the results by telephone with a healthcare professional, followed by 47.1% preferring a face-to-face meeting with a healthcare professional. Some form of verbal communication—whether face-to-face or by telephone—was preferred by 83.4% of women (85.0% when additionally considering that referral to a high-risk breast cancer center may lead to a face-to-face discussion). Of the 402 women who preferred to receive results by mailed letter or electronic communication, 245 (60.9%) also wanted some form of verbal communication (i.e., face-to-face or by telephone; 255 [63.4%] when additionally considering referral to a high-risk breast cancer center). Moreover, 77.2% of women preferred having detailed information about their exact risk estimate.

### Preferred Level of Detail for Risk Communication

**Fig. 3:**
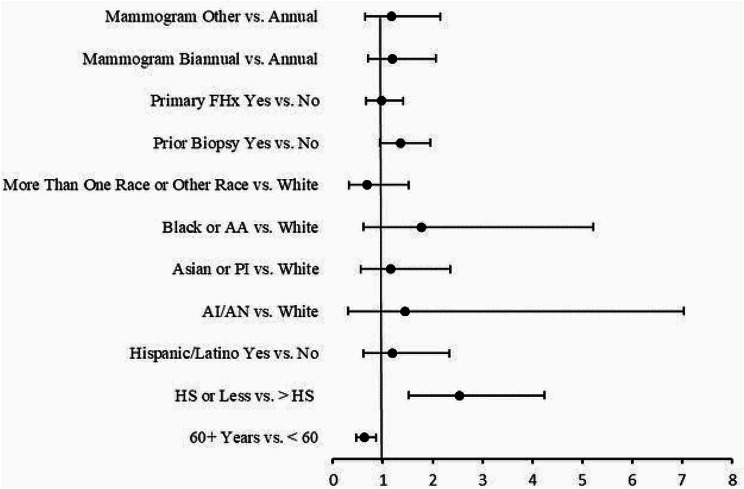
Forest plot of the odds ratio and 95% confidence intervals of factors predicting for knowing exact breast cancer for the entire sample (*N*=1037). Abbreviations: FHx, Family History; AA, African American; PI, Pacific Islander; AI/AN, American Indian or Alaskan Native; HS, High School

Results from the multivariable logistic regression analyses (see Fig. 3) show that women 60 years of age and older were significantly less likely to prefer exact estimates of their risk compared to women under the age of 60 (OR 0, 95% CI 0.5, 0.9; *p* = 0.003). Women with greater than a high school education were significantly more likely to prefer exact risk estimates, compared to women with a high school degree or less (OR 2.5; 95% CI 1.5, 4, *p* < 0.001). Based on the full sample, we did not observe significant differences in preferences for detailed risk estimates by race, ethnicity, prior breast biopsy, primary family history of breast cancer, or mammography frequency. Results from the supplementary analysis excluding women who were ever provided their risk estimates show similar patterns by age and education, as well as women with a history of breast biopsy being significantly more likely to prefer detailed risk information compared to those with no history of breast biopsy (OR 1.5; 95% CI 1.0, 2.3; *p* = 0.05).

## Discussion

This cross-sectional, quality improvement, survey study of women receiving a screening mammogram adds to the growing empirical evidence supporting women’s interest in and preferences for risk communication. Despite evolving guideline recommendations, only 13.2% of women in our study reported ever being told their personal breast cancer risk though nearly 73% of women were interested in knowing their breast cancer risk. Differences in interest in knowing breast cancer risk were observed by family history and race. We also found that women, if identified as increased risk, would prefer to receive their exact risk estimate verbally (i.e., phone or face-to-face) from a health care professional, though differences in preferences were observed by age and education.

Though multifactorial, women’s interest in knowing their breast cancer risk is key to successful implementation of BCRA programs [23]. Similar to prior studies [37], the majority of women in our study expressed a strong interest in knowing their estimated lifetime risk of breast cancer, aligning with the growing emphasis on shared decision-making and women’s autonomy in modern medicine [38, 39]. However, we also identified groups who may be less interested in their breast cancer risk including women identifying as “more than one race or other race” in our study. Not much exists in the current literature to explain this phenomenon. One potential explanation could be the low proportion of our sample identifying as “more than once race or other race” (*n* = 47/1119, 4.3%). However, we did not see differences among other race groups with similarly lower proportions. Higher levels of perceived risk have been associated with a higher degree of willingness to undergo BCRA in prior studies and is consistent with health behavior theories including the Health Belief Model and Protection Motivation Theory [24, 26, 40]. This could also explain increased interest among women with a primary family history of breast cancer, where their experiences and knowledge might influence their perceived breast cancer risk.

Contrary a prior study [41], we found that women 60 years of age or older were less likely to prefer exact information about their cancer risk. While breast cancer risk increases with age so do complications from other chronic conditions, which may explain lower preferences for exact breast cancer risk estimates. Additionally, we found that women with a high school degree or less were less likely to prefer exact information about their risk. This finding may be attributed to how the response options were presented, showing numeric risk estimates only. Low numeracy is pervasive, particularly among lower educated populations, and can impair risk communication as it is associated with difficulty in understanding and assessing risk-related information [42, 43]. Existing recommendations for risk communication suggest presenting information using a variety of formats including lay language, numerical (e.g., 20%; 1 out of 10) and pictorial information [44]. Additionally, decision support tools combining these recommendations with experience-based dynamic interfaces, such as games, has shown to significantly improve accuracy of breast cancer risk perceptions among high and low numeracy women [45]. However, qualitative analyses suggest that accurate risk perceptions alone are insufficient in the adoption of risk-appropriate breast cancer prevention strategies [45, 46].

Healthcare systems are encouraged to allow women to view and download their personal health records via electronic health record portals [47]. While leveraging such systems can support access to and personalization of care, our results support that women prefer verbal rather than written communications about their breast cancer risk [35, 37]. Combining written with verbal communication by clinicians has shown to be associated with greater awareness of one’s individual risk and greater adherence to guideline recommended care [48]. Yet, tailoring and communicating breast cancer risk to each women’s abilities and preferences can present significant challenges for clinicians and organizations, particularly in an era of increasing work volumes. Moreover, a recent study found that tailoring risk presentation formats to women’s preferences does not necessarily translate to improvements in risk comprehension [49]. Further research is needed to explore the feasibility, workflow challenges, and most effective formats for improving risk comprehension while aligning with women’s preferences [50].

This study has limitations. The cross-sectional nature of our study limits our ability to determine temporal causality of factors influencing interest and preferences for risk communication. Our study population was also limited to a single academic imaging center serving predominately educated, non-Hispanic, White women, thus limiting our ability to generalize findings to other settings. Despite the relative homogeneity of our sample, our large sample size allowed for detecting differences in interest and risk communication preferences by sociodemographic and clinical factors, critical for hypothesis generation. Specifically, we were able to detect lower levels of interest among women identifying as “more than one race or other race”, emphasizing the need for future studies to understand experiences and preferences of diverse populations. It is also important to note that the level of interest and preferences for knowing exact risk estimates may be overestimations since all women were recruited at the time of their screening mammogram and women demonstrating high levels of routine screening mammography. Future studies should assess acceptability of BCRA, including interest and willingness, among populations who lack access to or have not initiated screening, but may benefit from risk assessment and earlier screening.

## Conclusion

Risk assessment at the time of mammography screening has the potential to reach a wide audience, but ineffective risk communication may continue to undermine effective implementation and uptake of guideline recommended care. Our study adds to the growing empirical literature demonstrating that women are interested in and prefer to receive detailed breast cancer risk estimates verbally, though these preferences may differ by sociodemographic characteristics [35, 37]. These findings suggest that clinicians and organizations implementing risk assessment as part of routine mammography screening carefully consider methods for incorporating women’s communication preferences as part of an integrated, standardized workflow [15]. While combining written with verbal communication by clinicians was shown to be associated with greater awareness of one’s individual risk and greater adherence to supplemental MRI screening [48], presenting and discussing risk alone may not improve uptake of guideline recommended care [50, 51]. Providing risk information in conjunction with education on how to reduce risk has shown promising results [52], but education does not address other barriers that hinder uptake of recommended care including psychological distress, cost, transportation, and time [34, 53]. To this end, more research is needed to identify effective approaches to risk communication that also improve uptake on guideline recommended care. Additionally, our findings emphasize the need for more research to understand interest and preferences for risk communication among racially/ethnically diverse populations under the age of 40 who are not eligible for routine mammography screening but should undergo risk assessment in primary care settings.

### Electronic supplementary material

Below is the link to the electronic supplementary material.

Supplementary Material 1

## Data Availability

The datasets used and/or analyzed during the current study are available from the corresponding author on reasonable request.

## Abbreviations

MCA: Mayo Clinic Arizona
OR: Odds ratio
CI: Confidence Interval
