# Supplementary material for: Factors influencing U.S. women’s interest and preferences for breast cancer risk communication: a cross-sectional study from a large tertiary care breast imaging center
Source: BMC Womens Health. 2024 Jun 21;24:359. doi: 10.1186/s12905-024-03197-7 (PMC11191185; doi:10.1186/s12905-024-03197-7)
Supplement: Supplementary file 1 — Supplementary Material 1 [file 12905_2024_3197_MOESM1_ESM.pdf]

Breast Cancer Risk and Management Survey

We at the Mayo Clinic are seeking to know what women know about their own breast cancer risk. We would like to understand what type of information women prefer to receive about their breast cancer risk. The information from this survey will help us provide personalized breast care and ensure best patient outcomes.

We hope that you will answer all the questions. This survey is completely anonymous and your participation is voluntary.

This information may also be published in a medical journal to share knowledge with other health care professionals. If you have any questions regarding the study, please contact Danette Myers at myers.danette@mayo.edu. Thank you for helping us better understand the current needs of our breast cancer screening patients.

Breast Cancer Risk

1) Have you ever been provided an estimate of your risk of breast cancer?
- ◯ Yes
- ◯ No
- ◯ Not sure

2) I am interested in knowing my risk of developing breast cancer.
- ◯ Strongly agree
- ◯ Agree
- ◯ Neither agree nor disagree
- ◯ Disagree
- ◯ Strongly disagree

3) If your answer for question 2 is "Strongly agree or Strongly disagree," please explain why:

____

4) If you are found to be at HIGH risk of breast cancer, how would you prefer to receive the result of your estimated breast cancer risk? (check all that apply)
- ☐ Face-to-face meeting with the health professional who ordered the mammogram
- ☐ Telephone call from the health professional who ordered the mammogram
- ☐ Face-to-face meeting with the radiologist who interpreted your mammogram
- ☐ Telephone call from the radiologist who interpreted your mammogram
- ☐ Face-to-face meeting with a breast risk practitioner
- ☐ Telephone call from a breast risk practitioner
- ☐ Mailed letter accompanying your annual mammogram result
- ☐ Mailed letter separate from your mammogram result
- ☐ E-mailed copy separate from your mammogram result
- ☐ View the result through Patient Online Services (MyChart)
- ☐ Referral to a high-risk breast center

5) If you are considered at HIGH risk for breast cancer, how much detail would you want in the radiology report that is delivered to you?
- ◯ Less detailed (for example, "your calculated breast cancer risk was high and you may need further testing")
- ◯ Moderate detail (for example, "your calculated breast cancer risk was greater than 20% and you may need further testing")
- ◯ Very detailed (for example, "your calculated breast cancer risk was 26%, which is considered high risk, and you may need further testing")
- ◯ I would not like my risk to appear in my radiology report.

6) If you are found to be at AVERAGE risk, or are NOT at a high risk of breast cancer, how much detail would you want in the radiology report that is delivered to you?

- ◯ Less detailed (for example, "your calculated breast cancer risk was low and you need no further testing")
- ◯ Moderate detail (for example, "your calculated breast cancer risk was < 20% and you need no further testing")
- ◯ Very detailed (for example, "your calculated breast cancer risk was 16%, which is considered intermediate risk, and no further testing is needed")
- ◯ I would not like my risk to appear in my radiology report.

7) If you are considered at HIGH risk for breast cancer, your doctor may recommend you have additional tests to screen for breast cancer. How would you like to find out about that testing? (check all that apply)

- ☐ Face-to-face meeting with the health professional who ordered the mammogram
- ☐ Telephone call from the health professional who ordered the mammogram
- ☐ Face-to-face meeting with the radiologist who interpreted your mammogram
- ☐ Telephone call from the radiologist who interpreted your mammogram
- ☐ Face-to-face meeting with a breast risk practitioner
- ☐ Telephone call from a breast risk practitioner
- ☐ Mailed letter accompanying your annual mammogram result
- ☐ Mailed letter separate from your mammogram result
- ☐ E-mailed copy separate from your mammogram result
- ☐ View the result through Patient Online Services (MyChart)
- ☐ Referral to a high-risk breast center

Demographics

8) How old are you (years)?
- ◯ < 30
- ◯ 30-39
- ◯ 40-49
- ◯ 50-59
- ◯ 60-69
- ◯ 70-79
- ◯ ≥ 80

9) Select your annual household income:
- ◯ ≤ $20,000
- ◯ $20,001-$40,000
- ◯ $40,001-$65,000
- ◯ $65,001-$106,000
- ◯ ≥ $106,001

10) Select your highest level of education:
- ◯ Did not finish high school
- ◯ High school graduate or equal
- ◯ Some college
- ◯ Associate's and/or bachelor's degree
- ◯ Master's/doctorate and/or professional degree

11) Are you of Hispanic or Latino origin?
- ◯ Yes
- ◯ No

12) Do you consider yourself to be:
- ◯ African American, African descent, or black
- ◯ American Indian/Alaska Native
- ◯ Asian or Asian American
- ◯ Native Hawaiian or other Pacific Islander
- ◯ White or Caucasian
- ◯ More than one race
- ◯ Other race

13) If you selected "More than one race or Other race" please specify:

____

14) Do you have a primary care health provider?
- ◯ Yes
- ◯ No

Breast Care History

15) Do you have or have you had breast cancer?
- ◯ Yes
- ◯ No

16) Do you have a BRCA gene mutation (breast cancer BRCA1 or BRCA2) or other hereditary/genetic breast cancer mutation?
- ◯ Yes
- ◯ No

17) Have you had a breast biopsy?
- ◯ Yes
- ◯ No

18) If you have had a breast biopsy, how many?

____

19) Do you have a mother or sister who has had breast cancer?
- ◯ Yes
- ◯ No

20) At what age did you start having screening mammograms (years)?
- ◯ < 30
- ◯ 30-39
- ◯ 40-49
- ◯ 50-59
- ◯ 60-69
- ◯ ≥ 70

21) How often do you have a mammogram?
- ◯ Always once per year
- ◯ Often once per year
- ◯ Every other year
- ◯ Every few years
- ◯ Other
